# Systematic review of commercial artificial intelligence tools for the detection and volume quantification in intracerebral hemorrhage

**DOI:** 10.1007/s00330-025-11834-4

**Published:** 2025-07-24

**Authors:** Jana Sofie Weissflog, Emanuel J. Keller, Mitra L. Neymeyer, Andrea Morotti, Dar Dowlatshahi, Jawed Nawabi

**Affiliations:** 1 Department of Neuroradiology, Charité—Universitätsmedizin Berlin, Humboldt-Universität zu Berlin, Freie Universität Berlin, Berlin Institute of Health, Berlin, Germany; 2 Department of Anesthesiology and Operative Intensive Care Medicine, Charité—Universitätsmedizin Berlin, Humboldt-Universität zu Berlin, corporate member of Freie Universität Berlin, Berlin Institute of Health, Berlin, Germany; 3 Department of Neurosurgery, Charité—Universitätsmedizin Berlin, Humboldt-Universität zu Berlin, Freie Universität Berlin, Berlin Institute of Health, Berlin, Germany; 4 Department of clinical and experimental sciences, Neurology Clinic, University of Brescia, Brescia, Italy; 5 Neurology Unit, Department of Neurological Sciences and Vision, ASST-Spedali Civili, Brescia, Italy; 6 Department of Medicine (Neurology), University of Ottawa and Ottawa Hospital Research Institute, Ottawa, ON Canada

**Keywords:** Intracranial hemorrhages, Review [publication type], Artificial intelligence, Detection algorithms, Decision support systems (clinical)

## Abstract

**Objectives:**

This systematic review evaluates commercial imaging-based artificial intelligence (AI) software for intracerebral hemorrhage (ICH) detection and quantification.

**Materials and methods:**

A two-step approach was employed. (1) A systematic review, following PRISMA 2020 guidelines, searched PubMed and the Cochrane Library for studies on commercial AI tools for ICH imaging published between 1996 and March 2025, summarizing study designs, detection performance, and volume quantification metrics. (2) A cross-referencing process identified additional publications by consulting FDA and EUDAMED databases, AIforRadiology.com, and company disclosures through direct contact. Identified software was further evaluated in PubMed and the Cochrane Library to identify associated studies. Companies were contacted to verify publication records, regulatory approvals, validation studies, and clinical utilization.

**Results:**

From 2548 publications, 32 studies (2018–2023) met the inclusion criteria, covering 13 software solutions. Prospective designs were reported in 21.9%, with cohorts ranging from 102 to 58,321 scans. Detection performance demonstrated sensitivities of 68.2–99.7%, specificities of 83–97.7%, and accuracies of 85.3–99.16%. Volume quantification was assessed across seven tools, showing high correlations despite inconsistent metrics. Cross-referencing identified four additional tools lacking published studies. Among 19 tools identified, all were certified for ICH detection, 68.42% (13/19) for hematoma quantification—of these, 47.4% (9/19) had FDA certification only, two were pending approval, and one included hematoma expansion prediction. None disclosed internal validation studies.

**Conclusion:**

Commercial AI tools for ICH focus on detection and triage. Volume quantification tools remain limited, with variable performance and regulatory approval. Standardized protocols and greater transparency in validation are needed to enable meaningful comparisons.

**Key Points:**

***Question*** *Commercial AI tools for ICH detection and quantification lack standardized validation and comparative analysis, creating challenges for evaluation, comparison, and clinical integration*.

***Findings*** *Of 19 AI solutions identified, 13 had published studies. All supported ICH detection; six addressed volume quantification but varied in inconsistent designs and performance metrics*.

***Clinical relevance*** *Commercial AI tools for ICH are primarily validated for detection, while volume quantification remains less established. Variability in study designs and metrics limits comparability, underscoring the need for standardization to support clinical adoption*.

## Introduction

Advances in deep learning (DL) have significantly enhanced the capabilities of non-contrast computed tomography (NCCT) for detecting and estimating ICH volume, offering a substantial improvement over these methods [1–5] Capitalizing on these technological advancements, an array of Food and Drug Administration (FDA) and Conformité Européenne (CE)-certified software solutions have been introduced, offering fast and reliable detection capabilities and, increasingly, volume quantification to streamline patient triage and enhance radiological workflows. Nevertheless, there remains a substantial gap in the comparative and systematic evaluation of their analytical performance and regulatory profiles. To bridge this knowledge gap, we conducted a systematic review to aggregate and evaluate data on commercially available, tools for ICH detection and volume quantification on NCCT, utilizing DL technologies. Therefore, our systematic review adopts a two-fold approach: (1) conducting a systematic analysis of literature on commercial AI solutions for ICH, focusing on study design, methodology, clinical validation, performance metrics for detection efficacy, and volumetric segmentation; and (2) synthesizing regulatory aspects and technical profiles of commercially available tools, equipping clinicians with comprehensive data for informed decision-making.

## Methods

To enhance clarity and facilitate a better understanding of the terminology used during the review process, the term ‘intracerebral hemorrhage’ (ICH) has been used synonymously with ‘intraparenchymal hemorrhage,’ while other types of intracranial hemorrhage (ICRH) have been collectively summarized under the broader term ‘intracranial hemorrhage’ (ICRH), without further subclassification. This decision was made retrospectively, as most companies used the term ‘ICH’ interchangeably for intraparenchymal and other bleeding types. We specifically aimed to outline this distinction in as much detail and nuance as possible.

### Eligibility criteria

The following inclusion criteria were defined [1]: In order to identify commercially available software tools reported in the queried publications, either CE or FDA-approved tools were included [2]. Secondly, the software tools had to utilize DL-based technologies [3]. The software had to detect ICH on CT scans and/or provide volumetric segmentation output. Exclusion criteria were defined as the following [1]: software tools lacking current FDA- or CE-approval (e.g., outdated versions) [2]. Publications not indexed in PubMed or the Cochrane Library were also excluded.

### Search strategy

**Fig. 1:**
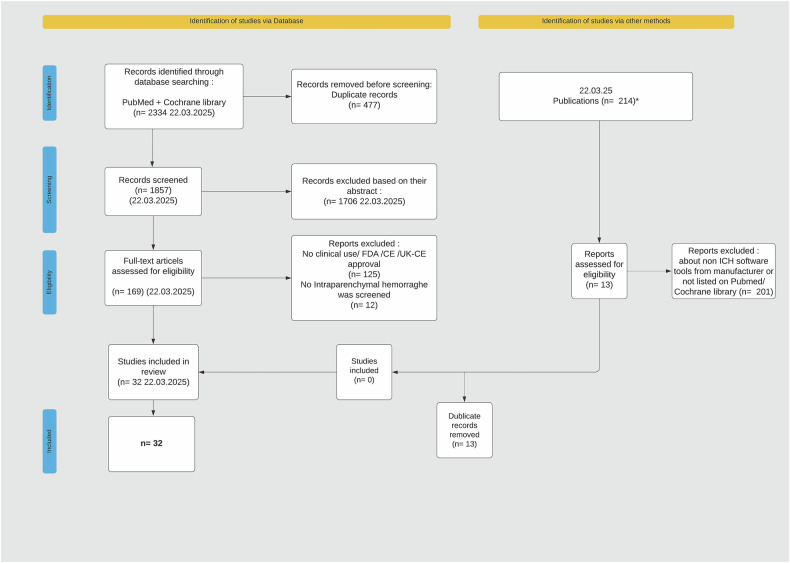
PRISMA 2020 flow diagram of the literature search. PRISMA 2020 flow diagram illustrating the study selection process. Studies were identified using PRISMA guidelines and a two-step approach. First, a database search was conducted using PubMed and Cochrane Library (left side). Second, a cross-referencing process identified additional publications by consulting the FDA and EUDAMED databases, AIforRadiology.com, and company disclosures through direct contact. Identified software was further evaluated in PubMed and the Cochrane Library to locate associated studies. The final set of studies was cross-checked with those identified in the database search to exclude duplicates. A total of 22 studies were included in the review. ^*^Six of nineteen manufacturers provided publication lists, and publications were identified from PubMed/Cochrane Library searches using manufacturer names and publication lists. FDA, Food and Drug Administration; CE, Conformité Européenne; UK-CE, United Kingdom Conformité Européenne

The data acquisition process was designed to ensure maximum data completeness and, therefore, included a two-step process. Figure 1 summarizes the first step. Therefore, a two-step systematic literature review was conducted in PubMed and the Cochrane Library on June 15, 2023, with updates on December 8, 2023, and March 22, 2025. The search adhered to the 2020 PRISMA guidelines [6] The following ten search combinations were used: ‘ICH Deep Learning algorithm’, ‘ICH Detection Software’, ‘ICH volumetric segmentation tool’, ‘ICH volumetric segmentation’, ‘ICH artificial intelligence’, ‘Intracerebral hemorrhage artificial intelligence’, ‘Intracerebral hemorrhage Deep learning algorithm’, ‘Intracerebral hemorrhage Detection Software’, ‘Intracerebral hemorrhage volumetric segmentation tool’, ‘Intracerebral hemorrhage volumetric segmentation’.

### Cross-referencing, synthesis of regulatory and technical profiles

**Fig. 2:**
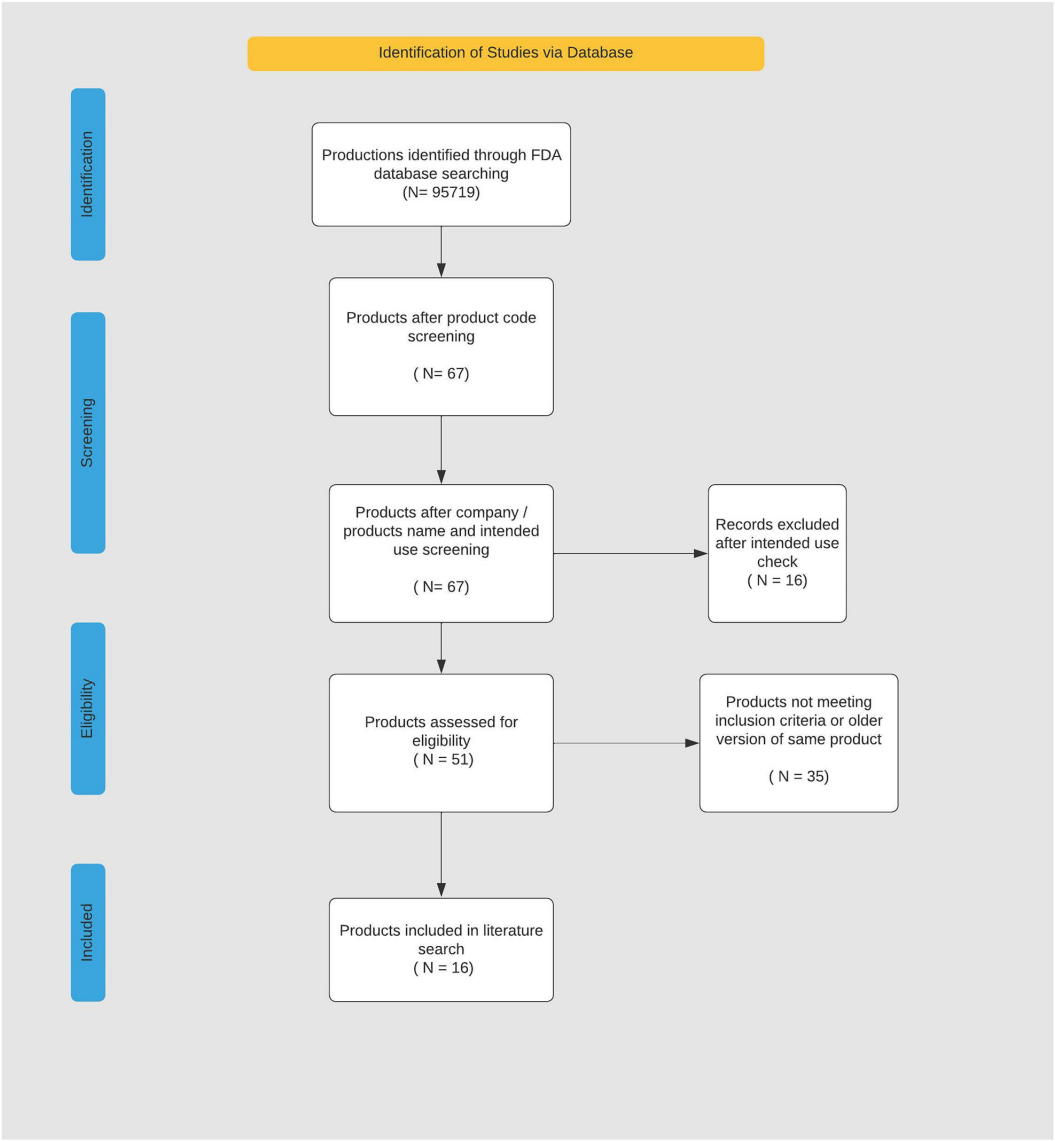
PRISMA 2020 flow diagram detailing the Step 2 literature search for FDA-approved AI-based ICH software solutions. PRISMA 2020 flow diagram illustrating the selection process for step 2. This step details the identification of software solutions through the FDA database search. A total of 90,834 records were identified, which were narrowed down using product code screening (*N* = 44). These records were then screened based on company name and intended use, resulting in 33 eligible products. Further exclusion of 11 records based on intended use and 22 records for not meeting inclusion criteria or being older versions of the same product led to the inclusion of 11 products in the literature search. Product code refers to the classification of approved devices according to function and indication, and *N* represents the number of software solutions. FDA, Food and Drug Administration; N, number of software solutions; CE, Conformité Européenne Certification; ICH, intracerebral hemorrhage; IVH, intraventricular hemorrhage; AI, artificial intelligence

To ensure a comprehensive evaluation of commercially available software tools, we performed a second step consisting of a multi-step cross-referencing and synthesis process, as illustrated in Fig. 2.

### FDA database cross-check analysis

The FDA database (covering 1996–2023) was systematically queried on September 6, 2023, and updated on March 22, 2025, to identify radiological AI tools meeting our eligibility criteria. The product code QAS was used as a filter, as it specifically refers to “Radiological Computer-Assisted Triage and Notification Software,” which includes tools that [7, 8]: (1) prioritize cases: identifying time-sensitive findings to expedite review; (2) issue triage notifications: alerting clinicians to critical imaging results requiring immediate attention (Supplementary Figs. 1 and 2).

### European database for medical devices (EUDAMED) database cross-check analysis

A similar search was conducted in the EUDAMED during the corresponding time periods [9]. However, no comprehensive results were retrieved. Upon formal inquiry, representatives of the European Commission and a Member of the European Parliament confirmed that the EUDAMED database currently lacks detailed public information regarding AI-enabled radiological tools. This was corroborated by an official communiqué dated October 19, 2023, from the Directorate-General for Health and Food Safety (Supplementary Figs. 3 and 4).

### AIforRadiology.com cross-check analysis

The AIforRadiology.com database was queried on November 13, 2023, and updated on March 22, 2025, to further validate and expand on the software tools identified from the FDA and EUDAMED databases [10].

### Company outreach cross-check, synthesis of regulatory and technical profiles

All identified companies were contacted via email on November 13, 2023, requesting detailed information on [11–23]: (1) publication records, (2) regulatory approval status of detection and volume quantification modules, (3) current clinical usage of their software, and (4) disclosure of any internal validation studies. Follow-up emails were sent on November 28, 2023, with a response deadline of December 5, 2023 (Supplementary Fig. 5). A second query was conducted on March 23, 2025, with an additional follow-up 7 days later. To ensure no studies were overlooked, the names of the identified companies and their software solutions were entered into PubMed and the Cochrane Library to identify any additional relevant publications.

### Verification of certification status

To ensure the accurate application of exclusion criteria, we further verified the regulatory approval status of each identified software program. Despite some prior publications noting FDA/CE certification, the certification status was re-validated through: (1) the FDA 510(k) database [24] and the EUDAMED database (when available) [25, 26].

### Study selection, data extraction, and quality assessment

#### Study selection

All retrieved publications were initially screened based on their abstracts by J.S.W. and M.L.N., independently applying the predefined inclusion and exclusion criteria. Studies meeting the criteria underwent full-text review, performed independently by J.S.W. and J.N., to confirm eligibility. In both stages, any discrepancies were resolved through consensus after joint re-evaluation.

#### Data extraction and quality assessment

**Fig. 3:**
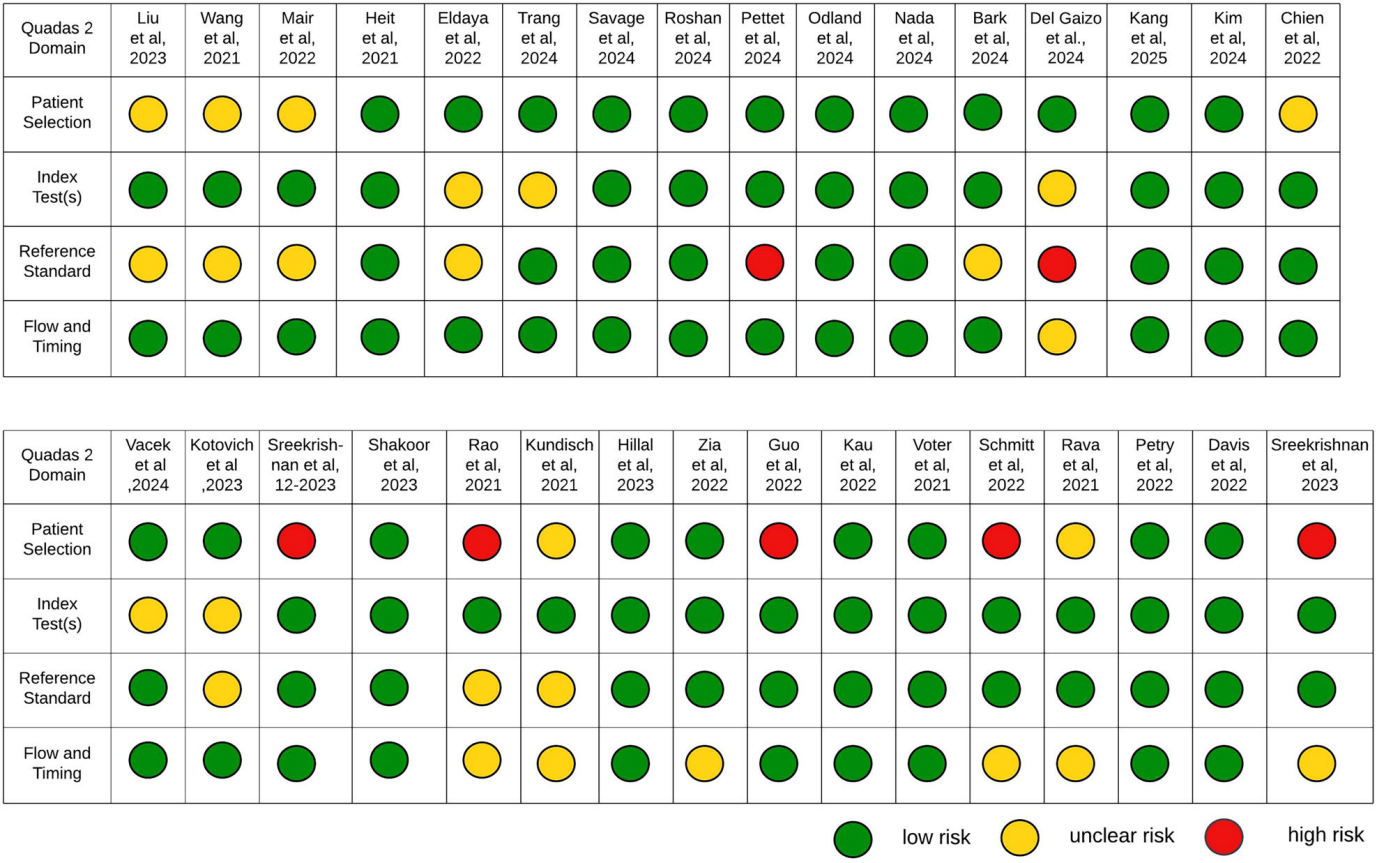
QUADAS-2 assessment of risk of bias and applicability concerns for the 22 included studies. Quality assessment of the 22 included studies, evaluated using the QUADAS-2 framework. The assessment focuses on four domains: patient selection, index test(s), reference standard, and flow and timing. Each domain is represented by colored circles to indicate risk of bias and applicability concerns: green denotes low risk of bias or concern, yellow indicates some concerns, and red represents high risk of bias or significant applicability concerns

Study details were extracted from relevant publications, including the company and software name, study title, authors, year, design (sample size, evidence level, dataset characteristics), performance metrics, and reference standards. Results are summarized in Table 1. Quality assessment was conducted using the Quality Assessment of Diagnostic Accuracy Studies tool (QUADAS-2) to evaluate diagnostic quality and risk of bias, independently reviewed by two authors (J.S.W. and J.N.), and presented in Fig. 3 and Supplementary Material [19].

**Table 1:** Synthesis of regulatory and technical profiles

Software solutions	Company name	Headquarters	FDA/CE approval status	Date of approval	Clinical implementation	Indication/function	Volume module approval status
Deep CT	Deep01 Limited https://www.deep01.com/products	New Taipei City, Taiwan	FDA: 510 (K) Class II K182875 CE-Certification Class l—MDD	10/2019 (first and last)	N/A	(1) ICRH detection (2) Triage function (3) Volume quantification	Approved
Aidoc ICRH	Aidoc Medical https://www.aidoc.com	Tel Aviv, Israel	FDA: 510 (K) Class II K250248 CE-Certification Class I—MDD	01/2018 (first) 02/2025 (last)	Yes	(1) ICRH detection (2) Triage function	N/A
Rapid ICH and Rapid Hyperdensity	Rapid AI (ISchemaView Inc.) https://www.rapidai.com	Redwood City, United States	FDA: 510 (K) Class II K221456 CE-Certification Class IIa—MDD	03/2020 (first) 12/2022 (last)	Yes	(1) ICRH detection (2) Triage function (3) Volume quantification	Approved
BioMind Diagnostic Support Software	Hanalytics https://biomind.ai/product	Singapore, Singapore	FDA: No CE-Certification Class IIa - MDD HD601504830001	07/2020	Yes	(3) Volume quantification (5) Hematoma expansion prediction (6) evaluating risk blood clot composition	Approved
qER	qure.ai http://qure.ai/	Mumbai, India	FDA: 510 (K) Class II K200921 CE-Certification Class 2b—MDR	06/2020 (first and last)	N/A	(1) ICRH detection (3) Volume quantification (4) ASPECTS scoring	Approved
Deepwise	Deepwise Website not available	Beijing, China	FDA: 510 (K) Class II K220910 CE-Certification N/A	03/2022 (first and last)	N/A	(3) Volume quantification	N/A
360 e-ASPECTS (Triage ICH)	Brainomix Limited https://www.brainomix.com/stroke/e-aspects/	Oxford, UK	FDA: 510 (K) Class II K231195 CE-Certification Class IIa—MDD	08/2023 (first and last)	Yes	(1) ICRH detection (2) Triage function (3) Volume quantification (4) ASPECTS scoring	Approved
InferRead CT Stroke	Infervision Medical Technology Co., Ltd. https://global.infervision.com/product/27.html	Beijing, China	FDA: 510 (K) Class II K211179 CE-Certification N/A	08/2021 (first and last)	N/A	(1) ICRH detection (3) Volume quantification	Approved
HALO	Nico.Lab B.V. https://www.nicolab.com/	Amsterdam, Netherlands	FDA: 510 (K) Class II K211788 CE-Certification Class IIb—MDR	08/2021 (first and last)	N/A	(1) ICRH detection (2) Triage function (3) Volume quantification	Approved Only CE: NOTIS-Number: 20213023
Viz ICH	Viz.ai https://www.viz.ai/	San Francisco, United States	FDA: 510 (K) Class II K232363 CE-Certification Class I—MDD	03/2020 (first) 02/2024 (last)	N/A	(1) ICRH detection (3) Volume quantification	Approval pending
CuraRad-ICH	Keya Medical https://www.keyamedical.com/curarad-ich/	Seattle, United States	FDA: 510 (K) Class II K192167 CE-Certification N/A	04/2020 (first and last)	N/A	(1) ICRH detection (2) Triage function	N/A
CINA ICH	AVICENNA.AI https://avicenna.ai/solutions/ai-tools-for-neuro/	La Ciotat, France	FDA: 510 (K) Class II K221716 CE-Certification Class IIb—MDR	11/2022 (first and last)	N/A	(1) ICRH detection (3) Volume quantification	Approval pending
Accipio lx	MaxQ Al Ltd. https://www.maxq.ai	Tel Aviv, Israel	FDA: 510 (K) Class II K201310 CE-Certification N/A	10/2018 (first) 08/2020 (last)	N/A	(1) ICRH detection	N/A
JLK ICH	JLK inc. https://jlkgroup.com/en/about/	Seoul, South Korea	FDA: 510 (K) Class II K243363 CE-Certification N/A	03/2025 (first and last)	N/A	(1) ICRH detection (2) Triage function (3) Volume quantification	Approved
NeuroICH	Neurocareai Inc. https://neurocare.ai/indications-for-use/	Frisco, United States	FDA: 510 (K) Class II K241719 CE-Certification N/A	07/2024 (first and last)	N/A	(1) ICRH detection (2) Triage function	N/A
uAI Easy Triage ICH	Shanghai United Imaging Intelligence Co. Ltd. https://www.uii-ai.com	Shanghai, China	FDA: 510 (K) Class II K242292 CE-Certification N/A	09/2024 (first and last)	N/A	(1) ICRH detection (2) Triage function (3) Volume quantification	Approved
Heuron ICH	Heuron Co. Ltd. https://iheuron.com/en/contents/neurovascular_ich.php	Seoul, South Korea	FDA: 510 (K) Class II K233247 CE-Certification N/A	05/2024 (first and last)	N/A	(1) ICRH detection (2) Triage function (3) Volume quantification	Approved
Hyper Insight—ICH	SK Inc. No website available	Bundang-Gu Seongnam-Si, South Korea	FDA: 510 (K) Class II K240353 CE-Certification N/A	01/2024 (first and last)	N/A	(1) ICRH detection (2) Triage function	N/A
HealthICH	Zebra Medical Vision Ltd. No website available	Shefayim, Israel	FDA: 510 (K) Class II CE-Certification N/A	06/2019 (first and last)	N/A	(1) ICRH detection (2) Triage function	N/A

Volume module status: single modules require separate approval even if the software as a whole is approved. The ASPECTS Score (Alberta Stroke Program Early CT Score) is a 10-point quantitative topographic CT scan score used for middle cerebral artery (MCA) stroke patients, with adjustments available for posterior circulation. MDR (Medical Device Regulation) is the current EU regulation that replaced the MDD (Medical Device Directive) as of May 2021. Manufacturers can no longer use the MDD to obtain CE certification for medical software. Products marked under the MDD may continue to be sold until May 2024, provided they have not expired or undergone significant changes. Update March 2025: all MDDs were not updated, and sale is currently suspended

*N/A* data not available, *CT* computed tomography, *ICRH* intracranial hemorrhage, *FDA* the American Food and Drug Administration, *CE* certification refers to Conformité Européenne Certification

#### Synthesis of regulatory status and status of clinical utilization

**Fig. 4:**
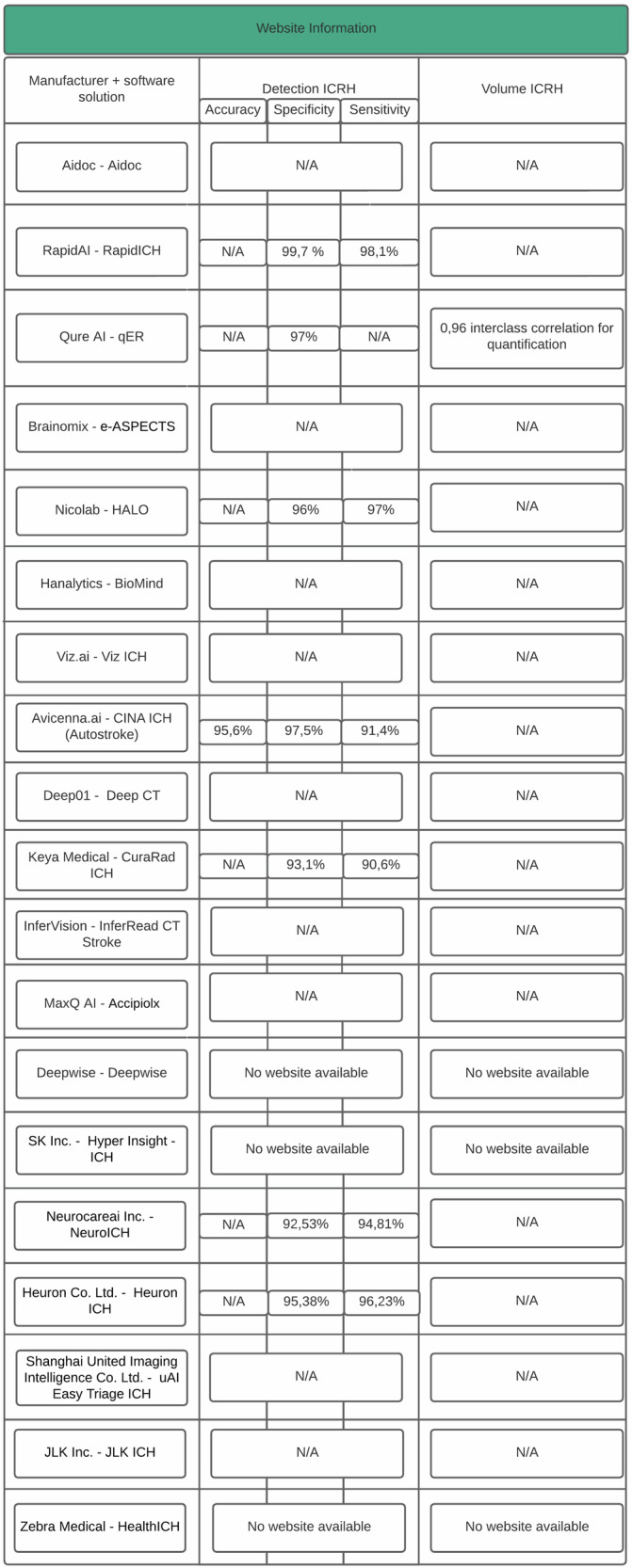
Overview of manufacturer-reported performance metrics for AI software solutions in ICRH detection and volume quantification. The table synthesizes information from manufacturer websites on AI software solutions for ICRH detection and volume quantification. Metrics include accuracy, specificity, and sensitivity for detection, as well as performance indicators for volume quantification when available. N/A indicates that the information was not disclosed on the respective website. For some software solutions, details on performance metrics for volume quantification were limited to statistical measures such as the Intraclass Correlation Coefficient (ICC). Detection metrics are provided for tools targeting ICRH, including subcategories such as intracerebral hemorrhage (ICH) and intraventricular hemorrhage (IVH), when applicable. “No Website Available” signifies the lack of accessible information for the corresponding manufacturer

A synthesis of regulatory status and clinical utilization is presented in Table 1, covering software solutions, company names, headquarters, FDA/CE approval status, approval dates, clinical implementation status, and module-specific details, including volume module approval. Performance metrics from company websites (as of April 19, 2025) are summarized in Fig. 4.

#### Statistics

No further statistical methods were applied. The Lucidchart application was employed for the visualization of data in our figures [27].

## Results

A systematic review of PubMed and Cochrane databases identified 32 eligible publications. An additional 13 publications were initially found through company-provided lists or searches for software solutions in the FDA and EUDAMED databases. After cross-referencing, these were excluded due to duplication.

A total of 32 studies were included, covering 13 different software solutions (Table 2). In the initial review round, 22 studies were included, representing nine different software solutions. Aidoc was the most frequently reported (9 studies, 41%), followed by e-ASPECTS (3 studies, 14%) and Rapid ICH (3 studies, 14%). Rapid Hyperdensity appeared in 1 study (4.5%), but combined with Rapid ICH, it represented 4 studies (18.2%). AutoStroke/CINA ICH, InferRead CT Stroke, BioMind, and Deep CT were each reported in 1 study (4.5%). While all tools addressed detection, only five included volume quantification. The second review round identified 10 additional studies from seven software solutions (Table 2). Aidoc again accounted for the largest share (3 studies, 30%). Newly reported software solutions not identified in the first round included Viz.AI ICH, JLK-ICH, Medical Insight, and HealthICH. Among these, Viz.AI was the only solution that supported volume quantification.

**Table 2:** Data extraction and summary of clinical validation studies for AI-based ICH detection and volume quantification software

Software solution (company)	Title, author, year	Sample size (scans)	Level of evidence	Dataset characteristics	ICRH detection	ICH detection	ICH volumetry	HE prediction	Other metrics/comments	Reference standard
qER (Company: Qure ai)	Accuracy of automated ICH volume measurement on NCCT: a Swedish Stroke Register cohort study, Hillal et al [37]	Total ICH *N* = 1649 ICH without IVH *N* = 891	III Retrospective cohort study	Multicenter (data from the “Riskstroke”; the Swedish quality register for stroke care)	NA	NA	Hematoma volumes were measured using three different volumetric methods: (1) manual segmentation of all ICRH; (2) fully automated segmentation of all ICRH (qER); and (3) ABC/2 method for ICH without IVH interrater agreement (1) ICC = 0.96 for manual segmentation vs qER (all ICH cases; *N* = 1581) (2) ICC = 0.97 for ABC/2 vs manual segmentation (ICH without IVH; *N* = 891) (3) ICC = 0.86 for ABC/2 vs qER (ICH without IVH; *N* = 891)	NA		GT segmentation was determined using the following two methods: (1) Manual segmentation by one resident with one year of neuroradiology experience (using sectra volume measurement tool)—with interrater agreement assessment with *N* = 500 scans with a senior neuroradiologist with more than 20 years of experience. (2) ABC/2 method
qER (Company: Qure ai)	A retrospective audit of an AI software for the detection of ICRH used by a teleradiology company in the United Kingdom Pettet et al [60]	Total head scans *N* = 1,315	III Retrospective	Multi-Center (data from a teleradiology practice)	PPA: 85.7% NPA: 94.3% PPV: 58.2% NPV : 98.6% Overall Agreement: 93.5%	N/A	N/A	N/A	**Comments:** (1) Overall agreement (~accuracy), PPA (~sensitivity), and negative percent agreement (NPA; ~specificity), to evaluate the agreement between AI and radiologists. (2) Agreement between the AI tool and the auditing radiologist was also quantified by Gwet’s AC113, Cohen’s kappa and prevalence, and bias-adjusted kappa (PABAK). (3) A multivariable logistic regression model was also fitted with age, sex, and the interaction term between age and sex as independent variables to investigate if there was any association of age and sex with incorrect AI results. **Other metrics:** (1) Agreement for presence/absence of ICH: 93.5% (95% CI, 92.1–94.8). Presence confirmed by AI in 96/112 cases (PPA: 85.7%, 95% CI, 77.8–91.6; NPA: 94.3%, 95% CI, 92.8–95.5). (16 FN outcomes (FN rate: 14.3%) and 69 FP outcome (FP rate: 5.7%)). (2) Almost perfect agreement for Gwert’s AC1 (0.92; 95% CI, 0.09–0.94); PABAK (0.87; 95% CI, 0.84–0.90); Cohen’s kappa: 0.66 (95% CI, 0.59–0.73). (3) No associations of age (*p* = 0.8), sex (*p* = 0.6), or the interaction term of age and sex (*p* = 0.8).	Single expert radiologist per scan (5 neuroradiologists reviewed *N* = 59 scans, 25 general radiologists reviewed *N* = 1256 scans—with an average experience of 13 years; general radiologists were all credentialled for CT head reporting) during an audit session of a teleradiology practice as part of their internal standard operating procedures. There was no delineation of cases between neuroradiologists and general radiologists. Cases were randomly selected for reviewing from the worklist (with evaluation of DICOM CT images first, followed by the radiology reports, and finally the AI-generated secondary capture images). Evaluation for true positive (TP), true negative (TN), false positive (FP), and false negative (FN) cases.
Deepwise (Company: N/A)	The signs of computer tomography (CT) combined with AI can indicate the correlation between the status of consciousness and primary brainstem hemorrhage of patients, Liu et al [49]	Total ICH *N* = 120 (All primary brainstem hemorrhage only)	III Retrospective cohort study	Singlecenter	NA	NA	NA	NA	**Comments:** The study explored the relationship between state of consciousness and the amount of bleeding volume. The application of the AI tool in this study was not for the purpose of validation; it served exclusively as a quantitative method for the measurement of blood volume.	NA
Rapid ICH (Company: RapidAI)	Automated cerebral hemorrhage detection using RAPID, Heit et al [36]	Total head CT scans *N* = 308 ICH *N* = 158	III Retrospective cohort study	Multicenter	Area under curve (AUC): NA PPV: 95.6% NPV: 95.3% Accuracy: NA Sensitivity: 95.6% Specificity: 95.3%	N/A	Volumetric quantification for ICH and IVH correlated with expert manual segmentation (Pearson correlation coefficient *r* = 0.983). The median absolute volume error (AVE) was 3 mL.	NA		Reference standard or GT segmentations were determined by consensus reading among three expert neuroradiologists.
Rapid ICH (Company: RapidAI)	Performance of automated RAPID ICRH detection in real-world practice: a single-institution experience, Eldaya et al [51]	Total head CT scans *N* = 307 ICRH *N* = 37	III Retrospective cohort study	Singlecenter	AUC: NA PPV: 44.7% NPV: 98.7% Accuracy: 85.3% Sensitivity: 91.9% Specificity: 84.4%	N/A	NA	NA	**Other metrics:** RAPID ICH detection was compared with the interpretation of a reference standard. When neuroradiologist and rapid ICH worked together, scores improved significantly, but Rapid ICH alone has a low PPV. Accuracy was 98.7% to the reference standard.	Reference standard was determined by a board-certified or board-eligible neuroradiologist, or in cases of discrepancy, adjudicated by a consensus panel of 3 neuroradiologists (with 3–24 years of experience as neuroradiologists)
Rapid hyperdensity (Company: RapidAI)	Automated cerebral hemorrhage volume calculation and stability detection using automated software, Sreekrishnan et al [35]	Total ICH *N* = 127	III Retrospective cohort study	Multicenter (data from two randomized clinical trials)	NA	NA	Automated rapid hyperdensity software was used to accurately measure ICH volume across repeated imaging. The two modalities were highly correlated: → baseline volume was highly correlated (*r* = 0.994, *p* < 0.001) → follow-up volume was highly correlated (*r* = 0,941, *p* < 0.001); with the ability of the AI software to detect ICH expansion with a Sensitivity of 94.12% and Specificity 97.27%. **Comments:** Volume quantification of AI software was compared with that of a neuroimaging expert.	NA		GT segmentation was determined by one expert (using medical image processing, analysis, and visualization (MIPAV) software)
Rapid ICH (Company: RapidAI)	Decreasing false-positive detection of ICRH using RAPID ICH 3, Seekrishnan [34]	Total head CT scans *N* = 881 ICRH *N* = 463	III Retrospective cohort study	Multi-center	AUC: NA PPV: 99.56% NPV: 97.65% Accuracy: NA Sensitivity: 97.84% Specificity: 99.52%	N/A	Review of the 10 false-negative cases indicated the AI software failed to identify 10 small ICHs with a median volume of 1.60 mL (IQR: 0.55–2.42). No ICH above 4 mL was missed by the AI software. **Comments:** Only ICH above 0.04 mL were included.	NA	**Other metrics:** The positive and negative likelihood ratios for ICH detection were similarly favorable at 204.49 and 0.02, respectively. Mean processing time was < 40 s.	The reference standard was determined by consensus review of at least two neuroradiology experts.
Deep CT (Company: Deep01)	Pilot report for ICRH detection with DL implanted head computed tomography images at Emergency Department, Chien et al [50]	Total head CT scans *N* = 238 ICRH *N* = N/A	III Prospective scale feasibility study (cohort)	Multicenter (data from Taiwanese National Health Insurance system)	AUC: NA PPV: 96.43% NPV: 99.52% Accuracy: 99.16 Sensitivity: 96.43% Specificity: 99.52%	N/A	NA	NA	**Other metrics/ Comments:** The study was conducted in a two-stage approach. The second stage of the study was a non-controlled, retrospective pilot clinical trial. Total *N* = 2999; with *N* = 2811 non-ICH and *N* = 188 ICH. Implementation of DeepCT resulted in shortened length of stay (LOS) in the emergency department (*p* = 0.0232) in patients with ICH.	NA
BioMind (Company: Hanalytics)	External validation study on the value of DL algorithm for the prediction of HE from noncontrast CT scans, Guo et al [55]	Total ICH *N* = 102	III Retrospective cohort study	Singlecenter	NA	NA	NA	AUC: NA PPV: NA NPV: NA Accuracy: 75.5% Sensitivity: 80.0%% Specificity: 73.6%	**Other metrics:** The sensitivity, specificity, and accuracy of HE prediction in the AI group were higher than those in the doctor group, *p* < 0.05. The AI diagnosis time (2.8 ± 0.3 s) and the doctors’ diagnosis time (11.7 ± 0.3 s) were both significantly shorter than the gold standard diagnosis time (14.5 ± 8.8 h) (*p* < 0.05), AI diagnosis time was significantly shorter than that of doctors (*p* < 0.05). **Comments:** Of note, the reported gold standard diagnosis time was not further defined. In the initial study, before the commercial implementation, the proposed model achieved a sensitivity and specificity of 89.3% and 81.1% for hematoma expansion.^1^ Teng et al (2021) Artificial intelligence (AI) can effectively predict early hematoma expansion of ICH analyzing noncontrast computed tomography image. Front Aging Neurosci 13:632138.	(1) GT segmentation was determined using the ABC/2 method by two experienced radiologists with 5 and 6 years of experience in IRCH imaging. (2) HE (increase of > 12.5 mL or 33%) was determined by two doctors and referred by a senior neuroradiologist (with 20 years of experience in IRCH imaging) to confirm the final diagnosis.
e-ASPECTS (Company; Brainomix)	Automated detection and segmentation of ICRH suspect hyperdensities in non-contrast-enhanced CT scans of acute stroke patients, Schmitt N et al [33]	Total stroke *N* = 160 ICRH *N* = 79 ICH *N* = 47	III Retrospective cohort study	Singlecenter	AUC: 90 PPV: NA NPV: NA Accuracy: NA Sensitivity: 91% Specificity: 89%	AUC: 93 PPV: NA NPV: NA Accuracy: NA Sensitivity: 98% Specificity: 89%	Interreader reliability of the quantitative ICH volume and the semi-automated reference had an ICC 0.98. The Dice Similarity Coefficient (DSC) was 0.82.	NA	**Other metrics:** Sensitivity and specificity for ICRH detection was computed for additional reader one (R1) of 0.99 and 0.98; and for reader two (R2) of 1.00 and 0.98. Interreader reliability for ICRH and ICH detection for the algorithm had an ICC of 0.80 and 0.84.	The reference standard or ground truth was determined by a board-certified neuroradiology consultant with more than 15 years of experience and full access to all clinical and radiological data.
e-ASPECTS (Company; Brainomix)	External Validation of e-ASPECTS Software for Interpreting Brain CT in Stroke, Mair et al [48]	Total stroke *N* = 4100 ICH *N* = 567	III Retrospective cohort study	Multicenter (data from seven national or international multicenter randomized controlled trials (RCTs) and two single-center prospective observational studies.)	AUC: NA PPV: 53% NPV: 98% Accuracy: 85% Sensitivity: 94% Specificity: 83%	N/A	NA	NA	**Comments:** The study was conducted in a two-stage approach. (1) The first stage Evaluated the performance of the software only in ischemic stroke patients which are not reported in this Table. (2) In a second stage a subanalysis was performed using an ischemic stroke cohort enriched with ICH positive and stroke mimics subjects creating a “Real-World Independent” cohort for the testing of e-ASPECTS” on which the presented data are based on. For ICH detection, e-ASPECTS had lower specificity, positive predictive value (PPV), and accuracy compared with experts, *p* < 0.0001. e-ASPECTS identified more false positive hemorrhage (14% vs < 1%) than experts.	The reference standard or ground truth was determined by a central expert panel (total 24 experts with crossover among studies, one expert report per scan), masked to follow-up imaging and most other clinical data.
e-ASPECTS (Company; Brainomix)	Evaluating AI software for delineating hemorrhage extent on CT brain imaging in stroke AI delineation of ICH on CT, Vacek [32]	Total ICH *N* = 628	III Retrospective cohort study	Multi-center (data from RITeS [Real-world Independent Testing of e-ASPECTS Software] cohort—a clinically representative sample of patients from 9 acute stroke studies.	NA	NA	In this study, the performance of the AI-tool was evaluated in the delineation ICH. Overall performance was excellent are good in 71% of cases. The quality of software delineation of ICH was better when fewer compartments were affected (*Z* = 3.61–6.27; *p* = 0.0063). Software delineation of ICH extent was more likely to be ‘excellent-good’ quality when lobar alone (OR = 1.56, 95% CI = 0.97–2.53). The software delineation was more likely to underestimate any IVH (OR = 0.56, 95% CI = 0.39–0.81, *p* = 0.002) or any extra-axial (OR = 0.41, 95% CI = 0.27–0.62, *p* < 0.001) extension. **Comments:** The cohort did not include patients with primary subarachnoid hemorrhage or hemorrhage secondary to venous sinus thrombosis or trauma.	NA		The reference standard was determined by a single expert (an expert panel conducted a prior imaging assessment for each study before its inclusion in the RITeS cohort).
AutoStroke CINA ICH (Company: Canon, software developer: Avicenna.ai)	Assessment of an AI Algorithm for Detection of ICRH, Rava et al [31]	Total ICRH and non-ICRH *n* = 302 ICRH *N* = 200 (ICH *N* = 181/200)	III Retrospective cohort study	Multi-center	AUC: NA PPV: 85% NPV: 98% Accuracy: 94% Sensitivity: 93% Specificity: 93%	AUC: NA PPV: 84% NPV: 98% Accuracy: 94% Sensitivity: 92% Specificity: 93%	95% Confidence intervals were calculated for ICRH and ICH volume, accuracy, sensitivity, specificity, PPV, NPV, F1 score, and Matthews correlation coefficients (MCC): (1) ICRH: 17.2 (mL) ± 2.7, accuracy of 0.94 ± 0.01, sensitivity of 0.93 ± 0.03, specificity of 0.93 ± 0.01, PPV of 0.85 ± 0.02, and NPV of 0.98 ± 0.01, F1 of 0.86 ± 0.03, MCC of 0.87 ± 0.02. (2) ICH: 19.1 (mL) ± 3.3, accuracy of 0.94 ± 0.01, sensitivity of 0.92 ± 0.03, specificity of 0.93 ± 0.02, PPV of 0.84 ± 0.03, and NPV of 0.98 ± 0.01, F1 of 0.85 ± 0.03, MCC 0.86 ± 0.02.	NA	**Other metrics:** A total of 95% (245 of 258) of ICH were correctly triaged, whereas 88.2% (90 of 102) of non-ICH cases were correctly classified as ICH negative.	(1) The reference standard was determined by consensus reading by two neuroradiologists. (2) GT segmentation was determined using an automated ICH segmentation tool within Vitrea (Vital Images, Minnetonka, Minnesota, USA). In the event of any erroneous ICH segmentation, manual contour adjustments were conducted.
CINA ICH (software developer: Avicienna.ai)	DL to Detect ICRH in a National Teleradiology Program and the Impact on Interpretation Time Del Gaizo et al [61]	Total head CT *N* = 58,321 (3383 excluded (5.48%) due to AI output error)	III Retrospective	Multi-Center	PPV: 21.1% NPV: 99.3% Accuracy: 91.7% Sensitivity: 75.6% Specificity: 92.1%	N/A	N/A	N/A	**Comments:** The system failed to process 5.5% of scans in a low prevalence (2.70%) environment. Reader interpretation times were longer when AI outputs included false positives or false negatives. Authors conclude that due to increased read times for falsely flagged exams; potential system inefficiencies in high-volume, low-prevalence settings may outweigh benefits of AI implementation. **Other metrics:** Falsely flagged studies by the AI solution led to lengthened radiologist read times and system inefficiencies (median read time increased 1 min 14 s [*p* < 0.001] for examinations with false-positive findings and 1 min 5 s [*p* = 0.04] for examinations with false-negative findings).	Radiologist report used as ground truth; AI outputs cross-referenced by two senior radiologists
InferRead CT Stroke (company: InferVision)	Efficiency of a DL-based AI diagnostic system in spontaneous ICH volume measurement, Wang et al [30]	Total ICH *n* = 105 with IVH *N* = 49 and without IVH *N* = 56	III Retrospective cohort study	Single-center	NA	NA	Correlations and agreement analyses were used to analyze the differences in volume between three different segmentation methods: The AI tool, the manual segmentation, and the ABC/2 method by further subdividing the groups into two subgroups: (1) ICH without IVH: Pearson Correlation [*r*] = 0.994, 0.976, 0.974; *p* < 0.001; concordance correlation coefficient [CCC] = 0.993, 0.968, 0.967 given for Algorithm vs CTP, ABC/2 score vs CTP, Algorithm vs ABC/2 score. (2) ICH with IVH: The correlation and agreement between CTP and AI were strong (*r* = 0.996, *p* < 0.001; CCC = 0.996) only evaluated for Algorithm vs CTP.	NA		GT segmentations were determined using the following two methods, performed by two independent raters (neuroradiologists specialized in stroke): (1) manual segmentation in ICH with IVH (2) ABC/2 in ICH without IVH
Aidoc (Company: Aidoc)	DL algorithm in detecting ICRHs on emergency computed tomographies, Kundisch et al [40]	Total head CT scans *N* = 4946 ICRH *N* = 267	III Retrospective cohort study	Multi-center (data from a prospectively registered trial in Germany; DRKS-ID: DRKS00023593)	NA	NA	NA	NA	**Comments:** The study aimed to determine the number of additional ICHs detected by the AI algorithm and to evaluate reasons for erroneous results at a level I trauma center with teleradiology services. **Other metrics:** Complementing human expertise with AI resulted in a 12.2% increase in ICH detection. The AI algorithm overcalled 1.9% HCT. Many of the ICHs missed by the AI algorithm were located in the subarachnoid space (42.4%) and under the calvaria (48.5%).	The reference standard was determined by an independent radiologist. Discrepant cases were transferred to two independent neuroradiologists.
Aidoc (Company: Aidoc)	Diagnostic accuracy and failure mode analysis of a DL algorithm for the detection of ICRH, Voter et al [42]	Total head CT scans *N* = 3,605 ICRH *N* = 349	III Retrospective cohort study	Single-center	AUC: NA PPV: 81.3% NPV: 99.2% Accuracy: NA Sensitivity: 92.3% Specificity: 97.7%	N/A	NA	NA	**Comments:** The authors discussed concerns about the generalizability of the AI tool due to decreased diagnostic accuracy, alongside lower sensitivity and PPV.	The reference standard was determined by the attending neuroradiologist imaging report
Aidoc (Company: Aidoc)	Utility of AI tool as a prospective radiology peer reviewer—detection of unreported ICRH, Rao [47]	Total head CT scans *N* = 6,565 ICRH *N* = 980	III Retrospective cohort study	Multi-center	NA	NA	NA	NA	**Comments:** The study quantitatively evaluated the prevalence of ICH in cerebral scans initially reported as ICH-negative. **Other metrics:** The AI tool identified potential ICH in 28 scans, initially classified as negative for ICH (“negative-by-report” and “positive-by-AI solution”) in comparison to 16 cases reviewed by the reference standard (“negative-by-report” and “positive-by-consensus review”). The false-negative rate for radiologist-detected ICH was quantified at 1.6%. Anatomical distribution of missed diagnoses: The most frequently overlooked ICH locations were adjacent to the cerebral convexity and within the parafalcine regions.	The reference standard was determined by consensus review of three neuroradiologists of prior ICH-positive-flagged cases based on radiology reports.
Aidoc (Company: Aidoc)	FDA-approved DL software application vs radiologists with different levels of expertise: detection of ICRH in a retrospective single-center study, Kau et al [54]	Total head CT scans *N* = 2188 ICRH *N* = 214	II Prospective cohort study	Single-center	AUC: NA PPV: 69.5% NPV: 96.5% Accuracy: 94% Sensitivity: 68.2% Specificity: 96.8%	N/A	NA	NA	**Other metrics:** The interrater agreement between the resident and the certified radiologist was very high (κ = 0.89) and even higher (κ = 0.93) between the resident and the reference standard. The accuracy of the AI application was inferior to that of both the resident and the reference standard, *p* < 0.001.	The reference standard was determined by a resident (with 1 year of experience) and a subspecialized neuroradiologist (with 5 years of experience in neuroradiology)
Aidoc (Company: Aidoc)	Retrospective analysis and prospective validation of an AI-based software for ICRH detection at a high-volume trauma centre, Zia et al [41]	Total head CT scans *N* = 1,446 ICRH *N* = 180	II Prospective cohort study	Single-center	AUC: NA PPV: 81.8% NPV: 97.6% Accuracy: NA Sensitivity: 85.7% Specificity: 96.8%	N/A	NA	NA	**Other metrics:** The turn-around-time (TAT) was calculated for cases in the emergency department, inpatient and outpatient department. The difference in TAT for all cases and the inpatient cases were statistically significant (*p* = 0.017 and *p* = 0.003). The difference in TAT for the emergency and outpatient cohorts were not statistically significant (*p* = 0.59 and *p* = 0.07). **Comments:** A prior peer review of head CT scans was performed prior to the implementation of the AI tool to identify the department’s current miss-rate. The performance metrics were also calculated separately for emergency, inpatient, outpatient with being lowest in outpatient and highest in emergency an inpatient.	For the prospective review, the reference standard was determined by either consultant neuroradiologists, consultant radiologists, or senior radiology registrars.
Aidoc (Company: Aidoc)	Decreased hospital length of stay for ICH and PE after adoption of an AI-augmented radiological worklist triage system, Petry et al [53]	Total ICRH *N* = 1718	III Retrospective cohort study	Single-center	NA	NA	NA	NA	**Comments:** The study examined hospital length of stay (LOS) in different groups (ICRH, pulmonary embolism, and hip-fracture) either in pre- or post-implementation of the AI tool. **Other metrics:** For the ICH-diagnosed patients, a mean LOS of 10.92 and 9.62 days was observed for the pre-AI and post-AI time periods, respectively. The mean difference was 1.30 days (*p* value = 0.032), which resulted in an observed percentage decrease of 11.9%.	NA
Aidoc (Company: Aidoc)	Machine learning and improved quality metrics in acute ICRH by noncontrast computed tomography, Davis et al [52]	Total head CT scans *N* = 24,996	II Prospective cohort study	Single-center	NA	NA	NA	NA	**Other metrics:** The study evaluated the potential optimization of the clinical radiology workflow by analyzing the report turnaround time (RTAT) and length of stay (LOS) in the emergency department (ED). ED LOS decreased from 471 min to 425 min (*p* < 0.001) for patients without ICH, and from 527 to 491 min for those with ICH (*p* = 0.456). Inpatient LOS decreased from 18.4 to 15.8 days for those without ICH (*p* = 0.001) and 18.1 to 15.8 days for those with ICH (*p* = 0.02). **Comments:** A reference was made to prior work, which was not listed in PubMed, reporting: In an analysis of 7112 noncontrast head CTs the AI tool had a sensitivity of 95%, specificity of 99%, negative predictive value (NPV) of 98%, and PPV of 98%, with an overall accuracy of 98%.^1^ 1. Conference paper by Ojeda et al The utility of DL: evaluation of a convolutional neural network for detection of intracranial bleeds on non-contrast head computed tomography studies. DOI: 10.1117/12.2513167	NA
Aidoc (Company: Aidoc)	The impact on clinical outcomes after 1 year of implementation of an AI solution for the detection of ICRH, Kotovich [45]	Total ICRH *N* = 587	III Retrospective cohort study	Single-center	NA	NA	NA	NA	**Comments:** The study investigated whether the introduction of AI software had an impact on clinical outcome. Therefore, two time periods were examined, prior and post to the AI implementation: **Other metrics:** The 30- and 120-day all-cause mortality were significantly reduced in the post-AI group when compared to the pre-AI group (27.7% vs 17.5%; *p* = 0.004 and 31.8% vs 21.7%; *p* = 0.017, respectively). Modified Rankin Scale (mRS) at discharge was significantly reduced post-AI implementation (3.2 vs 2.8; *p* = 0.044).	NA
Aidoc (Company: Aidoc)	Application of AI centric workflows for evaluation of neuroradiology emergencies, Shakoor et al [46]	N/A	II Prospective cohort study	Multi-center	NA	NA	NA	NA	**Comments:** This pilot study assessed the workflow of the AI tool in in clinical practice of a tertiary level facility. Therefore, AI-centric interactions were assessed for radiologist and neuroradiologists in different stages of training and expertise. **Other metrics:** The median usage of AI-centric workflow was 28.8% in the prospective cohort and 21.8% in the retrospective cohort.	NA
Aidoc (Company: Aidoc)	External validation and performance analysis of a DL-based model for the detection of ICRH Nada et al [58]	Total head CT scans *N* = 5600 ICRH *N* = 1022 ICH *N* = 491	II Prospective	Single-center	AUC: 0.954 PPV: 82% NPV: 97% Accuracy: 94% Sensitivity: 89% Specificity: 96%	N/A	N/A	N/A	**Other metrics:** Secondary study aims: (1) Assess model performance across diverse patient risk factors - Unanalyzed scans (*N* = 1231): Incompatible protocol names, bone-only kernels, non-contrast CT missing, scan combination errors - False positives: dural calcifications, partial volume averaging, skull base streak artifacts - Lowest sensitivity: SDH (89.05%) - Gender effect: Male patients more likely labeled ICH+ (*p* < 0.001) - Trauma history: Higher ICH detection rate (*p* < 0.001) - Age correlation: Increased ICH likelihood with older age (*p* < 0.001) - Comorbidities: Operative history, hypertension associated with higher ICH likelihood (*p* < 0.001). Smoking reduced ICH likelihood and algorithm detection (*p* = 0.017). History of DM: Lower detection rate by AI (*p* = 0.905) (2) Gather user satisfaction feedback from radiologists of varying experience levels Majority of participants with positive experience; helpful (*N* = 10), extremely helpful (*N* = 5) Model use across all clinical settings for confirmation (10/15 participants). Image analysis time longer than expected (5/20 participants, 25%)	Four neuroradiologists independently reviewed each scan; discrepancies adjudicated by a senior neuroradiologist (served as ground truth)
Aidoc (Company: Aidoc)	Sociodemographic biases in a commercial AI model for ICRH detection, Trang et al [56]		III Retrospective	Multi-Center	PPV: 69.4%, NPV: 97.6% Accuracy: 93.1%, Sensitivity: 85.2% Specificity: N/A	N/A	N/A	N/A	**Comments:** The study evaluated sociodemographic bias in a commercial AI tool for ICH detection on head CT. **Other metrics:** (1) Significant bias in AI performance across race, sex, insurance, and socioeconomic status (e.g., PPV was lower in Black, female, and Medicaid patients) (2) AI model produced a higher false-positive rate (lower PPV) in specific sociodemographic groups, including female, Black, non-Hispanic patients, those with Medicaid or private insurance, and individuals from more socioeconomically deprived areas. While overall accuracy remained high, these disparities could lead to unnecessary alerts, increased diagnostic burden, and potential inequities in care for affected groups.	Initial diagnosis based on board-certified neuroradiologist reports; ambiguous cases re-reviewed by an experienced, blinded neuroradiologist
Aidoc (Company: Aidoc)	Prospective evaluation of AI triage of ICRH on noncontrast Head CT examinations Savage et al [44]	Total head CT scans *N* = 9954 (from patients *N* = 7371)	II Prospective, real-world diagnostic accuracy study	Single-center	PPV: 79.2% NPV: 96.9% Accuracy: 99.2% Sensitivity: 87.8% Specificity: 94.3%	N/A	N/A	N/A	**Comment:** Detection and turnaround time for radiologist performance with vs without AI. **Other metrics:** No significant improvement with AI in diagnostic metrics or turnaround time. Accuracy (99.5% vs 99.2%), sensitivity (98.6% vs 98.9%), PPV (99.0% vs 97.5%), NPV (99.7% vs 99.7%), specificity (99.8% vs 99.3%), turnaround time (147.1 vs 149.9 min). Specificity (*p* = 0.004), turnaround time (*p* = 0.11)	The reference standard (ground truth) for determining the presence or absence of ICRH was established through a structured, multi-level adjudication process. First, if the AI result and the radiology report were concordant (both positive or both negative), this agreement was accepted as the reference standard. For all discordant cases (*n* = 447), an independent review was conducted by one of six experienced board-certified neuroradiologists who were blinded to both the AI results and the original report. If this reviewer disagreed with the original classification, a final decision was made by a consensus of two additional senior radiologists. Additionally, a random 10% sample of concordant cases was also reviewed to verify reliability. This rigorous, blinded process aimed to ensure a high-quality and unbiased ground truth for evaluating AI performance.
Viz.ai ICH (Company: Viz.ai)	Revolutionizing ICRH Diagnosis: a retrospective analytical study of Viz.ai ICH for enhanced diagnostic accuracy, Roshan et al [28]	Total head CT scans *N* = 4203 Total ICRH *N* = 386 Total ICH *N* = 136	III Retrospective diagnostic accuracy study	Single-center; real-world clinical setting	PPV: 81% NPV: 99% Sensitivity: 85% Specificity: 98%	Acute* ICH: Sensitivity: 98% (*N* = 118) Subacute* ICH: Sensitivity: 69% (*N* = 16 cases) *Acute vs subacute classification not further specified in methodology	≤ 5 cc: 84% sensitivity (45 cases)^**^ > 5 cc: 99% sensitivity (78 cases)^**^ ^**^Based on calculations with the ABC/2 method	N/A	**Other metrics:** ICH subtype analysis for intraparenchymal, subarachnoid, subdural, intraventricular. Sensitivity improved with higher acuity and volume/size across all ICH subtypes: Acute vs subacute sensitivity: intraparenchymal—98% vs 69%; subarachnoid—92% vs 40%; intraventricular—83% vs 22%. False positive cases mainly meningiomas (14%) (Turn-around time reported < 2 min, however, not systematically evaluated in this study). **Comment:** Timing: Acute or subacute (intraparenchymal, subarachnoid, intraventricular); acute, subacute, chronic, or mixed (subdural). Volume categories: Intraventricular—minimal, moderate, large; subarachnoid—minimal, moderate, large, diffuse; intraparenchymal—< 5 cc, > 5 cc (measurement via planimetric AP × CC × TR/2; subdural size: < 10 mm, > 10 mm; volume classification based on neuroradiologist consensus)	All CT scans were independently reviewed by neuroradiologists blinded to the Viz.ai results. These readings served as the reference standard. In cases of disagreement (e.g., Viz.ai false positives), a senior neuroradiologist with 10 years of experience performed an additional review.
Viz.ai ICH (Company: Viz.ai)	Real-world evaluation of the accuracy of the Viz.AI automated ICRH volume calculation tool Odland et al [29]	Total ICRH *N* = 139 with spontaneous ICH > 10 mL within 72 h of symptom onset *N* = 58 with IVH	III Retrospective	Multi-center	IVH detection only PPV: 91.4% NPPV: 94.0% Sensitivity: 94.6% Specificity: 94.0%	N/A	Comparison between AI, modified ABC/2 (mABC/2), and semi-autonomous segmentation (SAS): (1) ICH AI vs SAS: - Absolute volume difference: 4.77 ± 4.06 mL, *p* < 0.01 - Absolute percent difference: 10.41 ± 7.92% - Pearson correlation coefficient (*R*²): 0.99 mABC/2 vs SAS: - Absolute volume difference 48.36 ± 9.48 mL, *p* < 0.01 - Absolute percent difference: 17.41 ± 14.81% - Pearson Correlation Coefficient (*R*²): 0.86 Volume estimates differ across all methods, *p* < 0.01 Overestimation rates for AI – 89.9%; mABC/2 – 54.7% Subgroup Analysis: (2) ICH with IVH AI vs SAS: Absolute volume difference: 3.26 ± 3.55 mL Pearson correlation coefficient (*R*²): 0.96 (3) Total ICRH - Absolute volume difference 5.27 ± 4.71 mL - Absolute percent difference 9.81 ± 5.84%	N/A	**Other metrics:** Time to volume: SAS: 424 ± 208 s; AI: 151 ± 49.7 s (*p* < 0.01) AI vs mABC/2: (1) Volume accuracy higher with AI for lobar (*p* < 0.01) and deep hemorrhages (*p* = 0.016). (2) Performance with secondary bleed types better for IVH (*p* < 0.01), SAH (*p* < 0.01), and in isolated hemorrhages (*p* < 0.01); no difference with vs without secondary bleeds overall (*p* = 0.13) Further AI sub-analysis: (1) No difference in volume estimation in the presence or absence of secondary bleed types (IVH, SAH, SDH) (*p* = 0.13) (2) No difference in volume estimation between lobar and deep hemorrhages (*p* = 0.65) **Comments:** Authors conclude, that (1) AI ist faster than SAS and more accurate than mABC/2. (2) Introduce a guide to understand the bias AI introduces (location, size, and secondary bleeding types).	Semi-autonomous segmentation (SAS) of ICH and IVH on open-source software (3D Slicer) by a trained student, adjudicated by a board-certified neurosurgeon (used as surrogate ground truth)
HealthICH (Company: Zebra Medical Vision)	Clinical Impact of an AI Decision Support System for Detection of ICRH in CT Scans, Bark et al [59]	Total head CT scans *N* = 2306 ICRH *N* = 278 ICH *N* = 65	III Retrospective	Single-center	PPV: 0.881	PPV: 0.823	N/A	N/A	**Comments:** The secondary aim was to analyze whether the AI model led to faster escalation of patient care or surgery within the first 24 h. **Other metrics:** Overall time benefit of 0.05-624 min (median = 13.37, IQR = 12.91). Only 17 of these 139 patients had a time benefit from AI detection of 30 min or more, and all 17 were routine scans. Of the 17 patients with > 30 min time benefit, none resulted in summoning the on-call neurosurgeon, none led to escalation of the level of care, and none led to emergency surgery. No time benefit from AI detection between groups with emergency surgery vs no surgery (*p* = 0.4611), or between groups with/without escalation of level of care (*p* = 0.1886). No correlation between time benefit from AI detection vs time to surgery (*p* = 0.498, *r*^2^ = 0.09619).	Final radiology report by board-certified neuroradiologist; unclear cases re-reviewed by experienced neuroradiologist
JLK-ICH (Company: JLK Inc.)	DL-assisted detection of ICRH: validation and impact on reader performance Kang et al [43]	Internal dataset: Total head CT scans *N* = 1370 ICRH *N* = 1144 No ICRH *N* = 226 External dataset: Total head CT scans *N* = 670 ICRH *N* = 219 (of these ICH *N* = 102)	II Retrospective	Single-Center External validation with multi-ethnic U.S. dataset* * from Segmed, Inc. (Stanford, CA, USA) and Gradient Health, Inc. (Durham, NC, USA).	For ICH, IVH, SDH, EDH AUC: 0.94 PPV 97.7 NPV: 93.0 Sensitivity: 98.7 Specificity: 88.5 External validation*: AUC: 0.96 Accuracy: 91.8% Sensitivity: 95.4% Specificity: 90.0% ^*^at fixed threshold of 0.5 mL	AUC: 0.94 PPV: 96.7 NPV: 99.0 Sensitivity: 99.7 Specificity: 88.5	N/A	N/A	**Comments:** (1) To evaluate the previously trained model of Kang DW, et al (SDH, SAH, small-lesion ≤ 5 mL) for detection across ICH types (IPH, EDH, SDH, SAH, IVH). (2) Using an external test dataset to evaluate generalizability. (3) Evaluation of diagnostic support for non-specialist physicians (non-board-certified neurologists or radiologists) in identifying ICH on head CT. **Other metrics:** Comparison between results of non-expert readers with and without AI assistance (*N* = 400): (1) AUC with AI assistance (0.97) is higher than without assistance (0.95); *p* = 0.009. (2) Sensitivity with AI assistance (91.6) is higher than without assistance (88.6); *p* < 0.001. Specificity was not different, *p* = 0.15. Sub-analysis: (1) Low-confidence cases exhibited lower accuracy compared to high-confidence cases; (2) Accuracy with AI assistance (80.1%) higher than without AI (71.2%); *p* = 0.028 vs high-confidence cases: accuracy with AI assistance (96.4%) higher than without AI (95.3% *p* = 0.069. (3) Low-confidence cases showed significant improvement when assisted by the DL algorithm: Accuracy with AI assistance (80.1%) increased from 71.2 without AI; *p* = 0.028.	For the South Korean dataset, two independent neurologists with 6–10 years of experience reviewed all cases; disagreements were additionally reviewed by a third reviewer with 11 years of experience, and the ground truth was established based on the majority vote of the three experts. For the U.S. dataset, discordant cases were reviewed and adjudicated by an experienced neuroradiologist with 15 years of experience.

*N/A* not available, *ICRH* intracranial hemorrhage, *ICH* intracerebral hemorrhage, *IVH* intraventricular hemorrhage, *AUC* area under curve, *PPV* positive predictive value, *NPV* negative predictive value, *ICC* intraclass correlation coefficient, *CCC* concordance correlation coefficient, *HE* hematoma expansion, *AI* artificial intelligence

Study characteristics varied widely. Sample sizes ranged from 102 to 24,996 total head scans on NCCT, with seven prospective studies (22%) and 25 retrospective (78%). Evidence levels were predominantly III, with seven rated at level II (22%). Hemorrhage types (ICH alone, with or without IVH) and datasets (single- or multicenter) varied. Detection performance metrics demonstrated sensitivities of 68–100%, specificities of 83–98%, and accuracies of 85–99% for ICH. Detection performance for ICRH, along with other key metrics and study-specific comments, is detailed separately in Table 1.

Volume quantification, reported for seven [28–37] tools, showed overall good to high correlations but inconsistent metrics. Predominantly, the Pearson Correlation Coefficient (PCC) and the Concordance Correlation Coefficient (CCC) emerged as principal measures for assessing agreement between automated and reference segmentations [30, 33, 35–37]. For instance, PCC values often exceeded 0.9, while CCC values frequently reported above 0.8 highlighted strong concordance. Other metrics, such as the Dice Similarity Coefficient (DSC) [33], were occasionally used to measure spatial overlap between the AI predicted volume estimates and the ground truth, as well as absolute and relative volume differences [28, 29, 36]. Most studies did not limit their reporting to a single metric, but rather included a combination of one to three of these performance measures.

Reference standards varied across studies and significantly influenced results. Manual segmentation by one or more neuroradiologists was the most commonly employed reference standard. For example, e-ASPECTS studies utilized single or multiple radiologist annotations, while Rapid ICH studies relied on automated segmentation verified through expert consensus. Some studies, such as gER and BioMind, incorporated additional methods, including the ABC/2 segmentation [38, 39]or semi-automated approaches alongside expert validation. More recent studies showed a shift toward clearer and more robust reference standard definitions, especially in detection-focused evaluations. In contrast, relatively early studies [40, 41] did not report the level of experience of the human raters, while others, relied on a single reviewer without detailing how quality or consistency was assessed [42]. More recent studies, including Kang et al [43] and Savage et al [44], implemented structured, multi-level adjudication processes involving experienced neuroradiologists and consensus protocols. However, this trend was not consistent across all recent publications. In particular, volumetric studies continued to show variability in reference standard quality. In Roshan et al [28], ground truth was defined via blinded neuroradiologist reads, but reproducibility was not assessed. In Odland et al [29], segmentation was performed semi-autonomously by a trained student and adjudicated by a neurosurgeon, again without validation of consistency. These findings highlight ongoing variability in reference standard quality, particularly for volumetric evaluation, reinforcing the need for standardized and transparent reporting protocols.

Supplementary material details study characteristics and quality assessments, with most studies showing minimal concerns about bias or applicability.

Cross-check analysis identified a total of 19 commercially available software solutions for ICH detection and/or volume quantification, with varying combinations of FDA and/or CE certification (Table 1). Among 19 tools evaluated, all were certified for ICH detection, and 68.4% (13/19) included functionality for hematoma volume quantification; however, a large proportion did not disclose performance metrics on their websites (Fig. 4). Of these, 47.4% (9/19) had FDA certification only, two had CE certification only (10.5%), two were pending approval (10.5%), one tool included hematoma expansion (HE) prediction (5.3%), and one did not disclose its regulatory status (5.3%). Manufacturer-reported metrics revealed substantial non-disclosure (Fig. 4), and no tool provided internal validation data. Eight software solutions lacked publicly available clinical studies: Infervision Medical Technology Co., Ltd., Nico.lab B.V., Keya Medical, MaxQ AI Ltd., NeuroCare.AI Inc., Shanghai United Imaging, Heuron Co., Ltd., and SK Inc.

## Discussion

### Key findings

This systematic review provides an overview of commercially available AI-based software solutions for ICH detection and volumetric analysis. We identified 32 PubMed or Cochrane-indexed studies covering 13 software solutions [28–37, 40–61], seven of which included volume quantification [32, 36–39, 42, 45, 46, 51]. Results revealed varying levels of evidence and robustness, with some tools showing high accuracy and others highlighting areas for improvement, particularly in detecting smaller hemorrhages [34, 50]. All included tools had FDA and/or CE approval status.

Cross-referencing identified a total of 19 solutions, of which six [19, 20, 62–65] lacked validation through publicly available studies, raising concerns about transparency and trust. Among the 19 tools, 68.4% (13/19) were certified for hematoma quantification [11, 13, 15–19, 21, 23, 38], with two [15, 55] having CE-only approval, two pending approval [12, 21, 31], and one including HE prediction [15, 55].

### Detection performance

Detection capabilities demonstrated commendable to excellent performance, with sensitivities of 68–100% [34, 54], specificities of 83–100% [42, 48], and accuracies of 85–99% [50, 51]. However, prospective studies showed reduced performance compared to retrospective designs, as seen in tools like RAPID ICH and e-ASPECTS. Collaborative use with clinicians improved diagnostic efficacy, highlighting the complementary role of AI [35, 48]. More recent studies from 2024 onward reveal a clear shift toward reporting more nuanced detection features, including multi-lesional classification (e.g., Kang et al and Roshan et al) [28, 43]. In some cases, tools were evaluated for their ability to differentiate secondary causes such as vascular malformations post-ICH (Kim et al), reflecting a broader clinical scope. Importantly, the enthusiasm around AI tools is now being balanced by the reporting of negative or more tempered findings: Del Gaizo et al highlighted workflow inefficiencies due to high false positive rates and a 5.5% failure rate in processing hemorrhagic cases [61]. Similarly, Savage et al [44]—the only prospective study beyond 2024—found no improvement in detection performance or turnaround time. Bark et al [59] further showed that even when detection was timely, it did not translate into faster escalation of treatment. These findings underscore that while diagnostic performance metrics may appear promising, real-world clinical impact remains uncertain and highly context-dependent. Finally, a growing number of studies are exploring interpretability and bias, as seen in Pettet et al [60] and Trang et al [56], who investigated associations between patient characteristics and AI misclassifications, focusing on topics of ethical and interpretable AI.

### Volume quantification

Volume quantification studies were smaller and relied on heterogeneous reference standards, including manual segmentations, ABC/2 formula estimates, and consensus panels [30, 32, 37, 39, 42, 45, 46, 51]. Metrics varied, with Pearson Correlation Coefficients (> 0.9) and CCC (> 0.8) being the most common [30, 33, 35, 37]. Some studies, such as RAPID, Brainomix, and Viz.AI also reported supplementary metrics like absolute or relative volume error [39] and Dice Similarity Coefficient (DSC = 0.82) [33]. Notably, AI tools struggled with smaller hemorrhages, with false negatives observed for lesions below 4 mL [36, 40].

### Additional functional capabilities

While IVH detection and acute HE prediction are clinically significant, only Biomind incorporated acute HE prediction, albeit with a small retrospective cohort [55]. No solutions offered distinct IVH quantification, underscoring an unmet need [66]. However, studies from the scientific community have demonstrated the feasibility of such approaches, highlighting potential for future developments [67]. Notably, Odland et al and Roshan et al [28, 29], using Viz.AI software, were among the first to report separate volume quantification for ICH and IVH as distinct lesion entities.

### Reference standard

The results highlight considerable variability in how reference standards are defined and implemented across commercial AI studies for ICH, with significant implications for performance comparability. While some recent studies have adopted more structured, multi-reader adjudication processes, particularly for detection tasks, many still rely on basic or poorly described ground truth definitions. Earlier studies often lacked disclosure of reviewer expertise or used single-rater designs without quality control, as seen in Kundisch et al, Zia et al, and Voter et al [40–42] Even among newer studies evaluating volume quantification, methodological limitations persist. Roshan et al [28], employed the modified ABC/2 method—an estimation tool known to overestimate volumes [68]. Similarly, Odland et al 2024 [29] used semi-automated methods without interrater or interrater agreement metrics. Notably, none of the reviewed studies reported formal quality assessments of segmentation. This lack of standardization and transparency raises concerns about the robustness of volume-related findings and reinforces the need for methodologically rigorous frameworks, as exemplified by more advanced designs like Nawabi et al [69].

### Regulatory and integration challenges

Regulatory oversight revealed inconsistencies, with some CE-certified tools nearing MDD (Medical Device Directive) expiration by 2024, as detailed in Table 1. Based on our analysis, none of these companies disclosed information regarding transition to the Medical Device Regulation (MDR), suggesting that these tools may no longer be eligible for continued placement on the EU market beyond existing MDD approvals. The market appears to be shifting focus, increasingly targeting the U.S. instead—a region with comparatively less stringent regulatory requirements and the added incentive of reimbursement pathways for clinically deployed AI tools [70]. Some tools marketed uncertified volume features, potentially misleading users unfamiliar with regulatory specifics. Reporting within clinical studies varied, with companies often referenced instead of specific software, reducing transparency and trust [31]. Manufacturer websites also showed inconsistencies in product details and performance metrics, with essential information on detection and volume quantification frequently missing or incomplete (Fig. 4), underscoring the need for standardized, transparent reporting to support informed clinical adoption.

### Limitations

This review’s limitations include potential exclusion of non-PubMed-indexed studies and challenges in verifying CE certifications due to EUDAMED’s non-compulsory registration. Proprietary metrics reported by companies, based on their internal studies, were excluded due to a lack of independent validation. Limitations of the review were impacted by the lack of standardized search protocols for CE certifications at the time, and the non-compulsory nature of EUDAMED registration.

## Conclusion

The current landscape of commercially available AI tools for ICH detection and volume quantification is promising, with potential to enhance triage, diagnostic accuracy, and patient outcomes. While detection tools are well-established, volume quantification remains a developing domain, with variability in performance metrics and a lack of standardized validation protocols. These issues hinder direct comparisons between tools and complicate clinicians’ decisions regarding integration into practice. Notably, some solutions lack publicly disclosed external clinical studies, further limiting transparency and independent evaluation. Recent studies from 2024 onward have begun to provide a more nuanced view, expanding the scope of evaluation to include multi-lesional detection (e.g., ICH, SAH, and SDH), interpretable and ethical AI concerns, and diagnostic limitations. Several prospective and real-world studies have reported limited or no impact on workflow efficiency, underscoring the need to move beyond performance metrics alone. Despite these advances, the number of high-quality prospective studies remains low. Addressing these gaps—through standardized validation protocols, improved disclosure practices, and more robust prospective evaluations—is essential for ensuring comparability, reinforcing trust in these tools, and facilitating their widespread clinical adoption to realize their full benefits for patient care.

## Supplementary information

ELECTRONIC SUPPLEMENTARY MATERIAL

## Abbreviations

AI: Artificial intelligence
AUC: Area under curve
CCC: Concordance correlation coefficient
CE: Conformité Européenne
CT: Computer tomography
DL: Deep learning
EUDAMED: European database for medical devices
FDA: Food and Drug Administration
HE: Hematoma expansion
ICC: Intraclass correlation coefficient
ICH: Intracerebral hemorrhage
ICRH: Intracranial hemorrhage
IVH: Intraventricular hemorrhage
NCCT: Non-contrast computed tomography
NPV: Negative predictive value
PPV: Positive predictive value
