# Supplementary material for: Systematic review of commercial artificial intelligence tools for the detection and volume quantification in intracerebral hemorrhage
Source: Eur Radiol. 2025 Jul 24;36(1):367–95. doi: 10.1007/s00330-025-11834-4 (PMC12711959; doi:10.1007/s00330-025-11834-4)
Supplement: Supplementary file 1 — ELECTRONIC SUPPLEMENTARY MATERIAL [file 330_2025_11834_MOESM1_ESM.pdf]

Systematic Review of Commercial Artificial Intelligence Tools for the Detection and Volume Quantification in Intracerebral Hemorrhage

ELECTRONIC SUPPLEMENTARY MATERIAL

Eur Radiol (2025) Weissflog JS, Keller EJ, Neymeyer ML, Morotti A, Dowlatshahi D, Nawabi J.

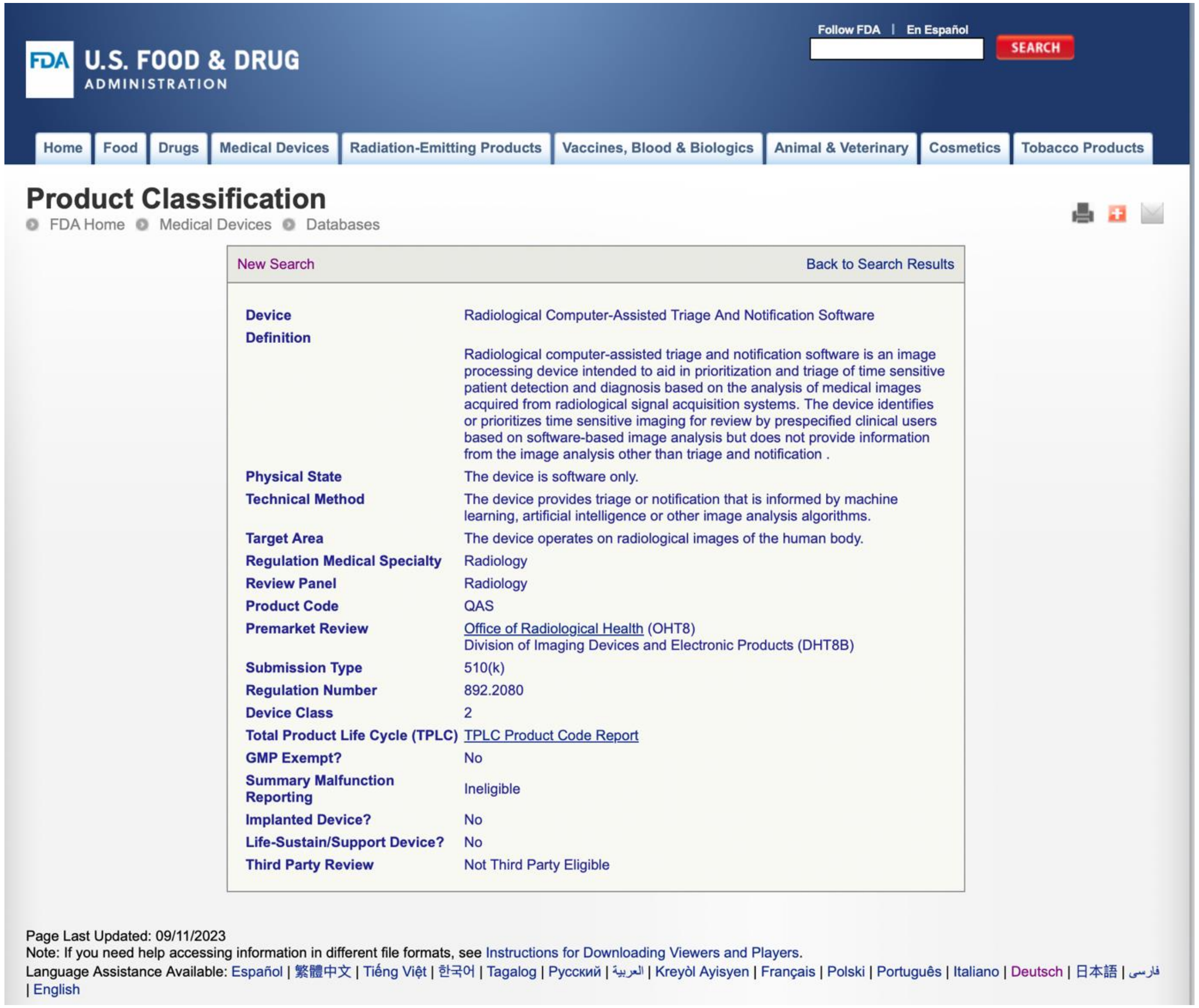

Follow FDA | En Español

SEARCH

U.S. FOOD & DRUG ADMINISTRATION

Home | Food | Drugs | Medical Devices | Radiation-Emitting Products | Vaccines, Blood & Biologics | Animal & Veterinary | Cosmetics | Tobacco Products

Product Classification

FDA Home · Medical Devices · Databases

New Search | Back to Search Results

**Device**	Radiological Computer-Assisted Triage And Notification Software
**Definition**	Radiological computer-assisted triage and notification software is an image processing device intended to aid in prioritization and triage of time sensitive patient detection and diagnosis based on the analysis of medical images acquired from radiological signal acquisition systems. The device identifies or prioritizes time sensitive imaging for review by prespecified clinical users based on software-based image analysis but does not provide information from the image analysis other than triage and notification .
**Physical State**	The device is software only.
**Technical Method**	The device provides triage or notification that is informed by machine learning, artificial intelligence or other image analysis algorithms.
**Target Area**	The device operates on radiological images of the human body.
**Regulation Medical Specialty**	Radiology
**Review Panel**	Radiology
**Product Code**	QAS
**Premarket Review**	Office of Radiological Health (OHT8)<br>Division of Imaging Devices and Electronic Products (DHT8B)
**Submission Type**	510(k)
**Regulation Number**	892.2080
**Device Class**	2
**Total Product Life Cycle (TPLC)**	TPLC Product Code Report
**GMP Exempt?**	No
**Summary Malfunction Reporting**	Ineligible
**Implanted Device?**	No
**Life-Sustain/Support Device?**	No
**Third Party Review**	Not Third Party Eligible

Page Last Updated: 09/11/2023
Note: If you need help accessing information in different file formats, see Instructions for Downloading Viewers and Players.
Language Assistance Available: Español | 繁體中文 | Tiếng Việt | 한국어 | Tagalog | Русский | العربية | Kreyòl Ayisyen | Français | Polski | Português | Italiano | Deutsch | 日本語 | فارسی | English

Figure 1 Product Classification QAS from FDA Website

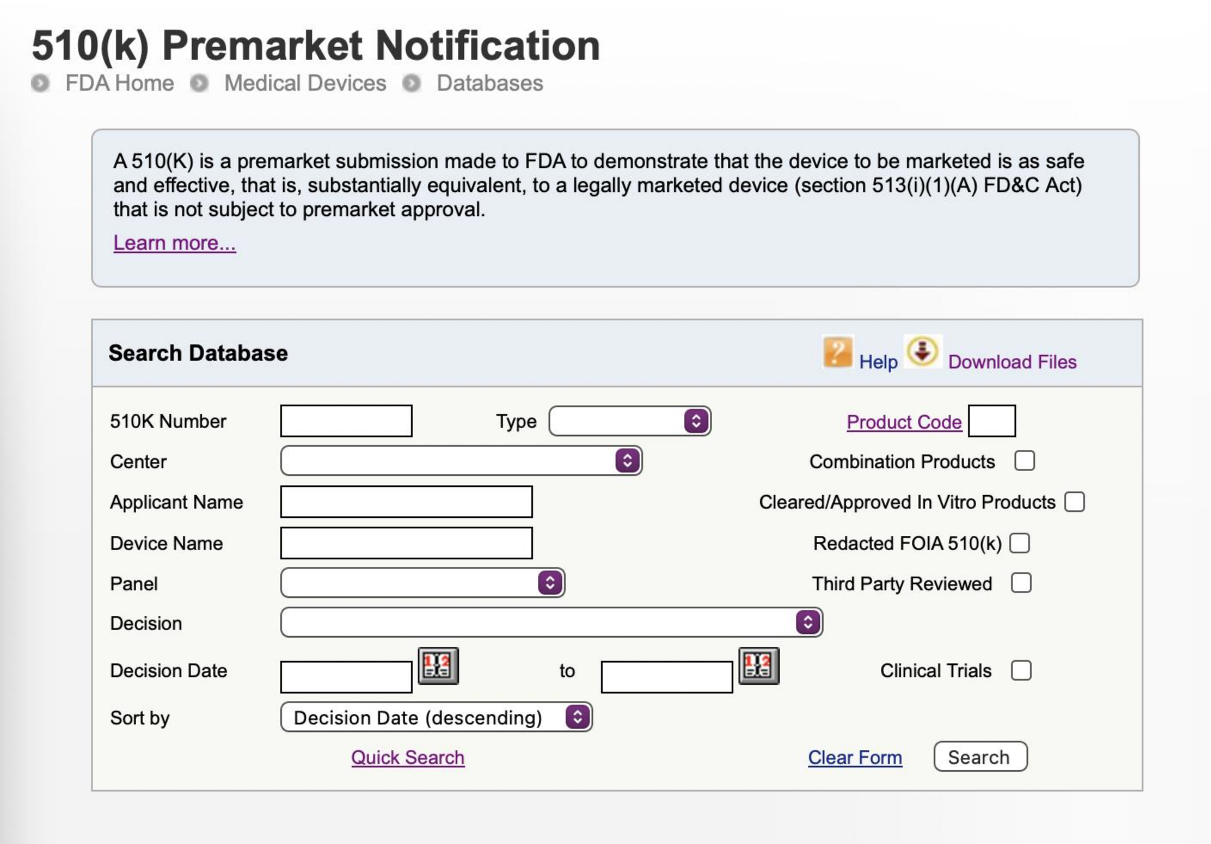

Figure 2 Screenshot of the utilized 510(k) Database Search tool

Von: [redacted]@europarl.europa.eu
Betreff: RE: [ext] Zulassungslisten Medizinische Produkte
Datum: 17. Oktober 2023 um 14:04
An: Weißflog, Jana Sofie jana-sofie.weissflog@charite.de

Sehr geehrte Frau Weißflog,

Vielen Dank für Ihre Email. Meines Wissens nach gibt es solch ein Dokument nicht. Es müsste tatsächlich die Kommission direkt kontaktiert werden mit der Frage. Aufgrund der starken Auslastung unseres Büros haben wir da gerade keine Kapazitäten zu und Sie müssten das selber machen. Evtl. kann diese Website helfen: https://health.ec.europa.eu/medical-devices-sector/new-regulations/contacts_en

Vielen Dank für Ihr Verständnis.

Mit freundlichen Grüßen

[redacted]

-----Original Message-----
From: Weißflog, Jana Sofie <jana-sofie.weissflog@charite.de>
Sent: 09 October 2023 12:00
To: [redacted]@europarl.europa.eu>
Subject: Zulassungslisten Medizinische Produkte

Sehr geehrtes Team [redacted]

[illegible]

Ich recherchiere aktuell zu einem großen Medizinischen Paper über CE und FDA Zertifizierte Software. (Im Bereich Neuroradiologie)

Die FDA hat auf ihrer Website Dateien zum Download für alle Medizin Produkte Zulassungen von 1996- jetzt. Das Suchtool EUDAMED das die europäische Kommission anbietet ist leider so unvollständig, dass das überprüfen einzelner Firmen schwierig ist. (Zum Beispiel Aldoc, man findet die Firma, aber nicht die Software mit der sicher vorhandenen CE-Zertifizierung). Aufgrund von Transparenz und Vollständigkeit habe ich großes Interesse an einer ähnlichen Datei wie die der FDA. Besteht irgend eine Möglichkeit eine ähnliche Datei zu erhalten? Für Rückfragen stehe ich jederzeit zur Verfügung.

Mit freundlichen Grüßen

Sofie Weißflog
Studentin der Humanmedizin
Doktorandin der Neuroradiologie Charite

Figure 3 Email correspondence with a member of the ***European Parliament*** *regarding the* ***availability and limitations of EUDAMED*** *(European Database on Medical Devices). In this exchange, the corresponding author (J.S. Weissflog) inquired whether a centralized, downloadable file—similar to the U.S. FDA's public listings—exists for CE-certified medical software devices. The response confirmed that, to the respondent's knowledge, such a document does not exist and that inquiries would need to be directed to the European Commission. Due to limited office capacity, the sender referred to the public EU medical device website instead. This highlights the ongoing challenges in accessing comprehensive EU regulatory data, particularly for academic and comparative research purposes.*

Von: **Europe Direct Contact Centre** EuropeDirectContactCentre@edcc.ec.europa.eu
Betreff: [ext] Ihre Europe Direct-Antwort Nr. #1258387
Datum: 19. Oktober 2023 um 17:14
An: jana-sofie.weissflog@charite.de

Sehr geehrte Frau Weissflog,

Vielen Dank für Ihre Anfrage an das Europe-Direct-Kontaktzentrum.

Wir haben die Generaldirektion Gesundheit und Lebensmittelsicherheit konsultiert. Nachstehend finden Sie die Antwort auf Ihre Frage.

„Wir haben nicht die Möglichkeit, von EUDAMED eine Datei mit der Liste aller registrierten Medizinprodukte zu erhalten.
Das System und das Verfahren für die Produktregistrierung sind in der EU (Kommission) und den USA (FDA) nicht identisch.
Die FDA verfügt über ihre GUDID-Datenbank mit Informationen zur einmaligen Produktkennung (UDI-DI), die von der FDA vorgelegt und genehmigt werden müssen und die eine Liste in einer Datei enthalten.

Auf der anderen Seite deckt EUDAMED auf EU-Ebene viel mehr ab als die UDI-DI, es befindet sich noch in der Entwicklung und wird nur auf freiwilliger Basis für die UDI/Produktregistrierung verwendet (was bedeutet, dass
erforderlich, bevor die Produktinformationen der Öffentlichkeit bereitgestellt werden.

Wir haben keine solche Datei mit UDI-/Produktdaten wie GUDID, aber es wird in Zukunft die Möglichkeit geben, Produktsuchergebnisse in einem XML-Format und/oder JSON-Format von der öffentlichen Website herunterzu

Wir hoffen, dass Ihnen diese Informationen weiterhelfen. Bitte kontaktieren Sie uns erneut, wenn Sie weitere Fragen zur Europäischen Union, ihren Aktivitäten oder Institutionen haben.

Add to europe mailing list: Yes

Nr. #1171007

Oben ist die Referenznummer angegeben , meine erneute Nachfrage:

Sehr geehrte Damen und Herren,

Mir ist die Datenbank EUDAMED durchaus bekannt, allerdings benötige ich eine Datei die Zulassungen von medizinischen Devices Zulassungen seit 1996 aufzeigt. Wie Sie in Ihrer Mail selber schreiben,

Mit freundlichen Grüßen
Jana Sofie Weißflog
Neuroradiologie Charite Mitte
+4917681250009

Mit freundlichen Grüßen
Europa Direkt Kontaktzentrum
Wir beantworten alle Fragen rund um

EUROPE DIRE
CONTACT CEN

00 800 6 7 8 9 10 11 • europedirect.e

Bitte beachten Sie, dass die von Europa Direkt zur Verfügung gestellten

Wir möchten Ihnen vier Fragen über die Anfrage stellen, die Eurc
Die gesamte Umfrage wird nur drei Minuten Ihrer Zeit in

Solidarität mit der Ukrai

Die EU und ihre internationalen Partner verurteilen Putins Überfall auf die Uk
Unterstützung – auch bei der Weiterreise und Suche nach einem neuen Zuhause
finanziell und humanitär zur Seite. Russland und seine Verbündeten hingegen b
spüren.

Figure 4 Email correspondence with the ***Directorate-General for Health and Food Safety (DG SANTE)*** *via the* ***Europe Direct Contact Centre****. The inquiry, sent by the corresponding author (J.S. Weissflog), requested access to a downloadable dataset listing approved medical devices in the EU since 1996, comparable to the FDA's GUDID database in the United States. The response confirms that no such comprehensive, downloadable list currently exists within EUDAMED. The EU system differs from the U.S. approach, and while some product information may be made publicly available in the future via UDI-DI (Unique Device Identification – Device Identifier), the infrastructure is still under development and remains incomplete. At present, no official downloadable dataset (in XML/JSON or similar format) for EU-approved medical devices is available.*

Manufacturer	First Email contact	Publicationlists available	Second Email contact
**Rapid AI**	01.11.23	01.11.23	
**NICOLAB**	31.10.23	31.10.23	
**qER ai**	10.11.23	N/A	28.11 PhD supervisor contacted again via Email
**Aidoc**	13.11.23	13.11.23	
**Brainomix**	13.11.23	28.11.23	28.11.23
**CINA ICH**	13.11.23	29.11.23	28.11 via Email Michael Glasa (German customer service via Canon)
**VIZ ai**	13.11.23	N/A	28.11.23
**MaxQ AL**	13.11.23 via Website	N/A	13.11.23 via Website
**Cura-RAD ICH**	13.11.23	N/A	28.11.23
**Infer-Read CT Stroke**	13.11.23	N/A	28.11.23
**BioMind**	13.11.23 no response contact Formular , Default	N/A	28.11.23
**Deep CT**	13.11.23	N/A	28.11.23
**Deepwise**	No Website or email available	N/A	N/A
**JLK inc.**	23.03.25	24.03.25	
**Heuron Co. Ltd.**	23.03.25	N/A	N/A
**SK Inc.**	No Website or email available	N/A	N/A
**Neurocareai Inc.**	23.03.25	24.03.25	
**Shanghai United Imaging Intelligence Co. Ltd.**	23.03.25	N/A	N/A

Figure 5 Timeline of company outreach via email and corresponding responses regarding AI tool validation and regulatory details.
